# Machine learning in health financing: benefits, risks and regulatory needs

**DOI:** 10.2471/BLT.23.290333

**Published:** 2023-12-08

**Authors:** Inke Mathauer, Maarten Oranje

**Affiliations:** a Department of Health Financing, World Health Organization, 20 Avenue Appia, 1211 Geneva, Switzerland.

## Abstract

There is increasing use of machine learning for the health financing functions (revenue raising, pooling and purchasing), yet evidence lacks for its effects on the universal health coverage (UHC) objectives. This paper provides a synopsis of the use cases of machine learning and their potential benefits and risks. The assessment reveals that the various use cases of machine learning for health financing have the potential to affect all the UHC intermediate objectives – the equitable distribution of resources (both positively and negatively); efficiency (primarily positively); and transparency (both positively and negatively). There are also both positive and negative effects on all three UHC final goals, that is, utilization of health services in line with need, financial protection and quality care. When the use of machine learning facilitates or simplifies health financing tasks that are counterproductive to UHC objectives, there are various risks – for instance risk selection, cost reductions at the expense of quality care, reduced financial protection or over-surveillance. Whether the effects of using machine learning are positive or negative depends on how and for which purpose the technology is applied. Therefore, specific health financing guidance and regulations, particularly for (voluntary) health insurance, are needed. To inform the development of specific health financing guidance and regulation, we propose several key policy and research questions. To gain a better understanding of how machine learning affects health financing for UHC objectives, more systematic and rigorous research should accompany the application of machine learning.

## Résumé

Alors que l’apprentissage machine connaît un usage croissant pour les fonctions de financement de la santé (collecte de revenus, mise en commun et achat), les preuves manquent quant à ses effets sur les objectifs de la couverture sanitaire universelle (CSU). Ce document présente une synthèse des cas d’utilisation de l’apprentissage machine et de leurs avantages et risques potentiels. L’évaluation révèle que les différents cas d’utilisation de l’apprentissage machine pour le financement de la santé sont susceptibles d’affecter tous les objectifs intermédiaires de la CSU: la distribution équitable des ressources (à la fois positivement et négativement), l’efficacité (principalement positivement) et la transparence (à la fois positivement et négativement). Il existe également des effets positifs et négatifs sur les trois objectifs finaux de la CSU, à savoir l’utilisation des services de santé en fonction des besoins, la protection financière et la qualité des soins. Lorsque l’utilisation de l’apprentissage machine facilite ou simplifie des tâches de financement de la santé qui vont à l’encontre des objectifs de la CSU, différents risques se font jour, comme la sélection des risques, la réduction des coûts au détriment de la qualité des soins, la réduction de la protection financière ou la surveillance excessive. Les effets positifs ou négatifs de l’utilisation de l’apprentissage machine dépendent de la manière dont la technologie est appliquée et de l’objectif poursuivi. C’est pourquoi s’imposent des orientations et des réglementations spécifiques en matière de financement de la santé, en particulier pour l’assurance maladie (volontaire). Afin d’éclairer l’élaboration de telles orientations et réglementations, nous proposons plusieurs questions clés en matière de politique et de recherche. Pour mieux comprendre la façon dont l’apprentissage machine affecte le financement de la santé dans le cadre des objectifs de la CSU, une recherche plus systématique et plus rigoureuse devrait accompagner la mise en œuvre de l’apprentissage machine.

## Resumen

Aunque el uso del aprendizaje automático para las funciones de financiación sanitaria (recaudación de ingresos, mancomunación y compra) es cada vez mayor, no hay evidencias de sus efectos sobre los objetivos de la cobertura sanitaria universal (CSU). Este documento ofrece una sinopsis de los casos de uso del aprendizaje automático y sus posibles beneficios y riesgos. La evaluación revela que los diversos casos de uso del aprendizaje automático para la financiación sanitaria tienen el potencial de afectar a todos los objetivos intermedios de la CSU: la distribución equitativa de los recursos (tanto positiva como negativamente), la eficiencia (principalmente positiva) y la transparencia (tanto positiva como negativamente). También hay efectos positivos y negativos en los tres objetivos finales de la CSU, es decir, la utilización de los servicios sanitarios en función de las necesidades, la protección financiera y la atención de calidad. El uso del aprendizaje automático para facilitar o simplificar tareas de financiación sanitaria contraproducentes para los objetivos de la CSU plantea diversos riesgos, como la selección de riesgos, la reducción de costes a expensas de la calidad de la atención, la disminución de la protección financiera o el exceso de vigilancia. El carácter positivo o negativo de los efectos del aprendizaje automático depende de cómo y con qué fin se aplique la tecnología. Por lo tanto, se necesitan directrices y reglamentos específicos para la financiación sanitaria, en particular para los seguros de salud (voluntarios). Proponemos varias preguntas clave en materia de política e investigación para contribuir a la elaboración de directrices y reglamentos específicos sobre financiación sanitaria. A fin de comprender mejor cómo afecta el aprendizaje automático al logro de los objetivos de la CSU en el ámbito de la financiación sanitaria, la aplicación del aprendizaje automático debería ir acompañada de una investigación más sistemática y rigurosa.

## ملخص

رغم الاستخدام المتزايد للتعلم الآلي في أغراض تمويل الصحة (جمع الإيرادات والتجميع والشراء)، إلا أنه هناك حاجة لأدلة تثبت آثاره على أهداف التغطية الصحية الشاملة (UHC). تقدم هذه الورقة ملخصًا لحالات استخدام التعلم الآلي وفوائدها ومخاطرها المحتملة. يكشف التقييم أن حالات الاستخدام المختلفة للتعلم الآلي لأغراض تمويل الصحة، لديها القدرة على التأثير على جميع الأهداف الوسيطة للتغطية الصحية الشاملة - التوزيع العادل للموارد (سواء بشكل إيجابي أو سلبي)، والكفاءة (بشكل إيجابي في المقام الأول)، والشفافية (سواء بشكل إيجابي أو سلبي). هناك أيضًا آثار إيجابية وسلبية على جميع الأهداف النهائية الثلاثة للتغطية الصحية الشاملة، وهي الاستفادة من الخدمات الصحية بما يتماشى مع الحاجة، والحماية المالية، والرعاية عالية الجودة. عندما يؤدي استخدام التعلم الآلي إلى تسهيل أو تبسيط مهام التمويل الصحي التي تؤدي إلى نتائج عكسية لأهداف التغطية الصحية الشاملة، فإن هناك مخاطر مختلفة، على سبيل المثال تحديد المخاطر، أو خفض التكاليف على حساب الرعاية عالية الجودة، أو تقليل الحماية المحدودة، أو المراقبة الزائدة. ما إذا كانت آثار استخدام التعلم الآلي إيجابية أم سلبية، فإن ذلك يعتمد على كيفية تطبيق التكنولوجيا، والغرض منها. وعلى ذلك، فإن هناك حاجة إلى إرشادات ولوائح محددة للتمويل الصحي، وخاصة فيما يتعلق بالتأمين الصحي (الاختياري). للمساعدة في تطوير إرشادات ولوائح محددة للتمويل الصحي، فإننا نقترح العديد من الأسئلة المتعلقة بالسياسات الرئيسية والبحث. للحصول على فهم أفضل لكيفية تأثير التعلم الآلي على التمويل الصحي لتحقيق أهداف التغطية الصحية الشاملة، فإن تطبيق التعلم الآلي يجب أن يصاحبه بحث أكثر نظامية وصرامة.

## 摘要

机器学习在卫生筹资职能（提高收益、资金筹集和购买能力）中的应用越来越广泛，但缺乏证据表明其对全民健康覆盖 (UHC) 目标的影响。本文概述了机器学习的使用案例及其潜在优势和风险。评估表明，机器学习在卫生筹资各种使用案例的应用有可能影响到所有 UHC 的中间目标——资源的公平分配（既有积极影响，也有消极影响）、效率（主要是积极影响）和透明度（既有积极影响，也有消极影响）。对 UHC 的三个最终目标（即根据需要利用卫生服务、财政保障和优质护理）也都产生了积极和消极的影响。当机器学习的应用促进或简化了与 UHC 目标相悖的卫生筹资任务时，就会出现各种风险，例如风险选择、以牺牲优质护理为代价来降低成本、降低财政保障或过度监督。应用机器学习的影响是积极的还是消极的，取决于应用该技术的方式和目的。因此，需要制定具体的卫生筹资指南和法规，特别是针对（自愿）健康保险的。为了为具体卫生筹资指南和法规的制定提供信息，我们提出了几个关键的政策和研究问题。为了更好地了解机器学习会如何影响卫生筹资的 UHC 目标，应在应用机器学习的同时进行更系统和严格的研究。

## Резюме

Машинное обучение все чаще используется для выполнения функций финансирования здравоохранения (сбор доходов, объединение и закупки), однако данные о его влиянии на достижение целей всеобщего охвата населения услугами здравоохранения (ВОЗ) отсутствуют. В этом документе приводится краткий обзор примеров использования машинного обучения, их возможных преимуществ и рисков. По результатам оценки выяснилось, что различные варианты использования машинного обучения для финансирования здравоохранения способны повлиять на все промежуточные цели всеобщего охвата услугами здравоохранения: справедливое распределение ресурсов (как в положительную, так и в отрицательную сторону), эффективность (в основном в положительную сторону) и прозрачность (как в положительную, так и в отрицательную сторону). Кроме того, наблюдаются как положительные, так и отрицательные последствия для всех трех конечных целей всеобщего охвата услугами здравоохранения, а именно: использование медицинских услуг в соответствии с потребностями, финансовая защита и качественное обслуживание. Когда использование машинного обучения облегчает или упрощает задачи финансирования здравоохранения, которые противоречат целям всеобщего охвата услугами здравоохранения, возникают различные риски, например: отбор рисков, сокращение расходов за счет качества обслуживания, снижение финансовой защиты или чрезмерный надзор. Какими будут последствия использования машинного обучения, положительными или отрицательными, зависит от того, как и для каких целей применяется эта технология. Поэтому необходимы специальные руководства и нормативные акты по финансированию здравоохранения, особенно в отношении (добровольного) медицинского страхования. Для разработки конкретных рекомендаций и нормативных актов по финансированию здравоохранения предлагается несколько ключевых вопросов политики и исследований. Для более глубокого понимания того, как машинное обучение влияет на финансирование здравоохранения для достижения целей всеобщего охвата населения услугами здравоохранения, применение машинного обучения должно сопровождаться более систематическими и тщательными исследованиями.

## Introduction

Over the past 10 years, the number of publications on artificial intelligence and machine learning related to health financing tasks has markedly increased,^1^ in line with the trend of using artificial intelligence in digital health in general.^2^ Still, evidence lacks regarding the impact of artificial intelligence and machine learning on health financing and universal health coverage (UHC), as is true for the broader field of digital technologies for health financing.^3^^–^^5^

Many countries, regardless of income level, are undertaking reforms in their health financing system to progress towards and achieve the three UHC intermediate objectives (equitable use of resources, efficiency, and transparency and accountability) and three UHC final goals (utilization in line with need, financial protection and quality care).^6^^–^^10^ Transparency and accountability pertain to the responsibility of health providers and purchasing agencies to provide information on their performance to each other, citizens, patients and health ministries. In Box 1 we summarize the health financing functions and their related policy decisions and tasks. A detailed explanation of how these functions contribute to UHC objectives and goals can be found elsewhere.^12^^,^^13^

Box 1 **Definitions of revenue raising, pooling and purchasing**

- Revenue raising is the process of raising money to pay health system costs, for example through taxation and health insurance contributions. Revenue raising includes policy decisions and tasks related to the sources and levels of contributions, and the mechanisms to collect these funds.
- Pooling is the accumulation and aggregation of prepaid funds, so that the financial risk of having to pay for health care is shared by all members of the pool. Pooling includes decisions and tasks related to targeting and identification of population groups for subsidized health coverage, as well as risk adjustment or cross-subsidization across risk pools.
- Purchasing is the process of allocating these prepaid and pooled funds from purchasers to health-care providers. Purchasing includes decisions and tasks related to selection and contracting of providers, the provider payment methods and rates. Closely related to purchasing is benefits design, which concerns decisions on service and cost coverage, cost-sharing rates and exemptions.^11^

The World Health Organization (WHO) refers to artificial intelligence as “the ability of algorithms encoded in technology to learn from data so that they can perform automated tasks without every step in the process having to be programmed explicitly by a human.”^14^ Machine learning is one of the most prominent types of artificial intelligence. Machine learning uses statistical and mathematical modelling techniques, whereby algorithmic models are trained to recognize patterns and to make estimations or predictions based on these patterns without human interference.^14^

There are different types of machine learning. In supervised learning, the data used to train a model are labelled (the input and respective outcome variables are known) and the model derives a function from the training data that can predict outputs from new input data. Unsupervised learning does not use labelled data but involves the identification of hidden patterns in the data. In semi-supervised learning, a small amount of labelled (training) data is combined with a large amount of unlabelled data.^14^^,^^15^ Machine learning is particularly useful in the analysis of big data, which are data sets that are too complex for traditional data processing methods. Volume, variety and velocity are properties defining these data sets. Without the use of machine learning, health financing tasks supported by data analysis are undertaken by traditional statistical methods or actuarial analysis.

**Fig. 1:**
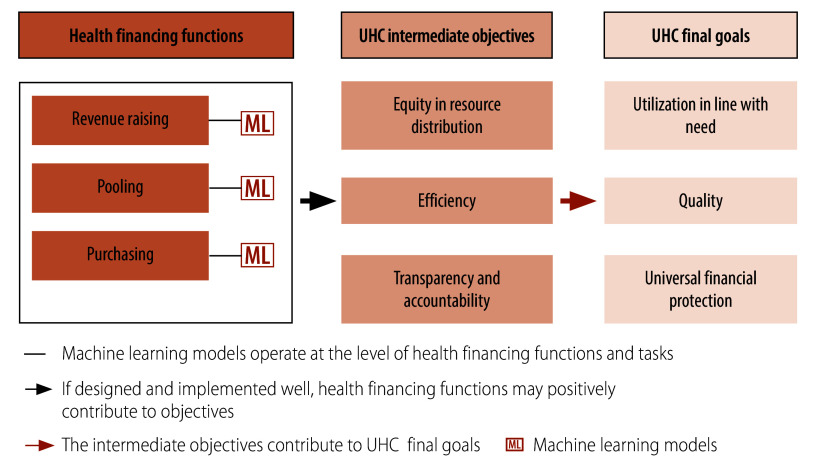
Linkages between machine learning models, health financing functions and UHC objectives and goals

Machine learning, like other digital technologies, may affect health financing and UHC objectives in various ways.^16^^,^^17^ Fig. 1 illustrates how digital technologies, specifically machine learning, interface with health financing functions and tasks, potentially enhancing intermediate and final UHC objectives. In an ideal scenario, machine learning would support, facilitate, enhance or simplify a health financing task or scheme, or inform health financing policy design that in itself is conducive to UHC. However, the use of machine learning can also have negative consequences. Inadequate design and/or flawed implementation of machine learning can harm a UHC-conducive health financing task, scheme or policy design. Equally concerning is the use of machine learning in ways that, while supporting, enhancing or simplifying health financing tasks or schemes, or informing policy design, may not be conducive to UHC a priori.^17^

UHC: universal health coverage.

Source: Adapted from World Health Organization.^17^

With the increasing use of machine learning in health financing, we aim to provide an overview of use cases in health financing, and the risks and benefits of using machine learning in this field. First, we provide a synopsis of the potential benefits and risks of using machine learning as a means to progress towards the intermediate and final UHC objectives. We then discuss notable policy implications and outline a range of key policy questions, with the aim of supporting country policy-makers, practitioners, researchers and development partners in their effective development and use of machine learning tools as well as artificial intelligence for UHC purposes.

## Machine learning in health financing

Building on existing literature,^1^ we have identified the main domains of health financing in which machine learning is currently being used in-country, or for which theoretical modelling exercises and model comparisons have been undertaken by researchers. Of note, there are only a few documented and published country use cases of machine learning for health financing. We have extracted the benefits and risks of using artificial intelligence in health financing from several review papers^1^^,^^18^^,^^19^ and have deductively derived the main implications in relation to UHC objectives. Table 1 presents the domains of health financing in which machine learning is used and the types of machine learning applied, as well as summarizes the risks and benefits. We discuss eight domains in health financing for which machine learning is used, cutting across all health financing functions, with an emphasis on purchasing. As illustrated in Table 1, both supervised and unsupervised models are being used. In-country uses and modelling exercises of machine learning for health financing show potential to affect intermediate UHC objectives. The efficiency objective is primarily positively affected by applying machine learning, while equitable distribution of resources and transparency are both positively and negatively affected. Furthermore, machine learning can have both positive and negative effects on all three UHC final goals.

**Table 1:** Uses of machine learning in health financing and their potential risks and benefits

Domain of use (function)	Type of application	Potential benefits	Risks
Prediction of health expenditure or health-care costs, also in relation to specific diseases or in relation to use by specific individuals or groups (revenue raising, purchasing)	Supervised learning (classification, variable selection, regression, prediction)	Through the revelation of hidden patterns in data (such as specific patient attributes or use patterns), more accurate cost predictions are possible (compared to traditional statistical methods), e.g. for specific diseases, for high-cost patients and for improved service tailoring (e.g. optimized disease management and prevention activities for specific population groups). These insights can contribute to better forecasting, more efficient spending, more equitable resource distribution and improved utilization in line with need.	Predictive analytics could be used for the exclusion of high-cost diseases and introduction of cost-reduction measures at the expense of financial protection and quality of care. Predictive analytics endangers a person's privacy, i.e. a person may not give consent for predictions to be made based on their personal data.
Assessment of health risks in a pool (i.e. calculating patients’ health risk scores, a metric to predict aspects of a patient’s future care (e.g. comparing costs, risk of hospitalization of a patient with the average) (pooling)	Supervised learning (regression, variable selection)	More precise risk scoring through machine learning supported data analysis can improve risk adjustment or risk equalization mechanisms and formulas, which serve to ensure equalized per capita allocations or expenditure that reflects differences in health risks and needs across different pools. More precise scoring can contribute to more efficiency and equitable allocation of resources.	More precise risk scoring may facilitate risk selection and the exclusion of high (cost) users or otherwise vulnerable groups from health insurance, or the increase of their premiums (based on medical underwriting). These practices could lead to further fragmentation of risk pools and hence reduced equity in resource distribution, as well as increased inequity in access to care and reduced financial protection.
Claims review and fraud detection (purchasing)	Mostly supervised learning (classification), some examples of unsupervised learning (clustering/outlier detection)	By identifying patterns, machine learning-supported claims review can improve and accelerate: preauthorization of patient care; claims adjudication (the checking of validity and eligibility); proactive identification of coding and billing errors before claims processing and payment; and detection and investigation of outliers and duplicate claims. These improvements can result in reduced claims processing time, reduced human resources needed for claims review, reduced administrative costs, less erroneous payments and higher overall efficiency. Prudence in claims preparation by providers can be enhanced (less gaming), leading to higher transparency and accountability of health providers and purchasing agencies in their claims management activities. Possible synergy between automated (machine learning-supported) claims review and review by humans (whereby the former can support the latter) might lead to less subjectivity and errors (more transparency of purchasing agencies in their claims review decisions).	Machine learning-supported claims management may enable more advanced, thus simplified, opportunities for surveillance of health service providers by the purchaser, potentially leading to a culture of fault-finding and mistrust among providers towards the purchaser. Machine learning-supported claims management could reduce the role of human judgment in claims review, which could lead to a lack of transparency and explicability of outcomes of purchasing agencies, and as a result of algorithmic bias, could lead to discrimination against certain population groups.
Design or revision of provider payments through claims analysis (purchasing)	No information on the applied types of machine learning	The use of machine learning results can provide more granular and more accurate insights to inform policy decisions on: provider payment methods and rates; and quality improvement, based on comparison of treatment practices across providers. Such policy decisions can result in improved efficiency, more equitable distribution of resources and more equitable access to care, financial protection and improved quality of care.	Machine learning-supported data analysis may suffer from algorithmic bias and/or use of insufficient or unrepresentative data. Bias could lead to unintended, distorted outcomes, including unequal treatment of providers and potential market distortions. Lack of explicability of the working and outcomes of machine learning to health-care providers could erode trust.
Provider performance and contract monitoring by purchasers (purchasing)	No information on the applied types of machine learning	Automated performance monitoring and contract monitoring of providers by the purchaser can result in resource and time savings and elimination of human errors.	
Design of benefits and access conditions based on claims analysis (purchasing)	Unsupervised learning (clustering); also supervised learning (regression/ prediction)	Machine learning-based claims analysis can provide more granular insights on patient needs and expenditure trends to inform policy decisions on premiums and co-insurance rates (as cost determinants become clearer) and health needs-oriented benefits design. Using these insights for policy decisions can result in increased efficiency, more equitable distribution of resources and equitable access to care, quality of care and responsiveness to patient preferences and needs, and financial protection.	Machine learning-supported data analysis may facilitate algorithmic bias and/or use of insufficient or unrepresentative data for policy decisions, potentially leading to unintended and distorted outcomes, including discrimination against individuals or population groups and increasing inequities in resource distribution. Lack of explicability of the working and outcomes of machine learning to beneficiaries could erode trust of people in social protection schemes and programmes. Combination of data from multiple databases can increase opportunities for surveillance and lack of privacy.
Identification of beneficiaries for targeting policies (pooling and purchasing)	Supervised learning (classification, variable selection, regression); unsupervised learning (clustering)	Precision and efficiency can be increased in targeting processes for poor and/or vulnerable beneficiaries of government-funded health coverage or cash transfer programmes, as well as in setting differentiated and tailored cost-sharing policies, for example by applying machine learning to multiple, combined data sets. Such improved precision can lead to higher administrative efficiency and more equitable financial protection.	
Beneficiary enrolment (pooling)	Supervised learning (classification)	Identity confirmation, identification of beneficiaries and timely enrolment can be improved, which can reduce coverage gaps (exclusion errors) and increase financial protection. Validation of data entry in beneficiary databases, e.g. through identity confirmation and authentication support processes, improves data quality. As a result, fraud, abuse, inclusion errors and unnecessary data collection can be reduced.	

Source: Compilation of various review papers.^1^^,^^18^^,^^19^

As the use of machine learning in health financing is rapidly evolving, we are aware that this overview of use cases and benefits and risks is not exhaustive nor complete. The use cases covered here comprise all WHO regions. In the rapid literature review^1^ covering 38 studies, for instance, 58% (22 studies) of these are based on data from high-income countries, with more than half (12 studies) coming from the United States of America, while the rest used data from low- and middle-income countries.

There are assumingly further machine learning applications in health financing, for instance in voluntary health insurance. These applications appear to be neither systematically documented nor published yet, and would eventually complement this overview.

Additionally, machine learning can be applied to areas adjacent to health financing, such as procurement, supplier selection, and the optimization of hospital (financial) management practices. These include improving diagnosis-related grouping systems, implementing cost-containment measures, and optimizing resource allocation to increase the financial efficiency of hospitals.^1^ We also observe that many of the identified machine learning use cases have been applications that support health insurance-related tasks, which suggests to us that potential uses of machine learning in health financing have not yet been sufficiently explored for other health coverage schemes.

### Benefits

The main advantages of machine learning lie in the higher accuracy and enhanced speed in generating results in comparison to traditional statistical methods or conventional prediction models, as data processing and analysis can be automated. Increased accuracy and speed may also result in (administrative) cost reductions for the different health financing actors, such as purchasers, providers and stewards, particularly in relation to claims management and fraud detection,^19^^,^^20^ as practically applied in an increasing number of countries.^21^^–^^24^ Also numerous theoretical modelling exercises show these benefits. For instance, a study using public health insurance data from Ghana designed and evaluated algorithmic models that served to classify health insurance claims into legitimate and fraudulent claims, demonstrating improved accuracy in classification while also considerably decreasing processing time.^25^ In another instance, data from a performance-based payment programme for health centres in Zambia were used to test various machine learning techniques to identify which health centres require verification of their reported performance. The model showed that targeted verification, as opposed to undifferentiated verification of all providers, might provide cost savings by reducing the number of fraudulent claims and unnecessary verification.^26^

Machine learning can also be useful in the prediction of health-care expenditure, the targeting and identification of beneficiaries and the selection of providers. For example, several supervised machine learning techniques were tested to predict the development of Swiss patients’ health-care costs and to identify factors contributing to their increase or decrease. These findings could enable policy-makers to improve resource allocation planning, optimizing health service delivery, for example through targeted disease management programmes for high-cost patients.^27^

Machine learning models may also reveal patterns in the available data, such as specific patient attributes, that otherwise may remain hidden. For example, a study using data from a nationwide health survey in Portugal applied unsupervised learning to identify clusters within the uninsured population, finding three segments with distinct sociodemographic characteristics and health-related needs. This information could be used to inform benefits design and policies to improve access to health care.^28^ Finally, machine learning could bring an advantage in technical or administrative processes where there are potential conflicts of interest or rent-seeking implications. Since human-to-human interactions are minimized, there is less room for arbitrary or discriminatory practices, for instance in relation to identifying households eligible for subsidization of health coverage.

### Risks

In addition to the possible benefits, there are also critical risks. A generic problem, beyond the health financing sector, is the use of insufficient or unrepresentative data to train algorithms as well as bias in their design; exacerbating inequities or even leading to exclusion of certain population groups from benefits.^29^

Other ethical issues, which can create negative outcomes if not properly addressed, relate to (i) enduring digital divides between different population groups (in terms of income, gender, remoteness); (ii) use of poor-quality data when no other data is available; (iii) mixed effects of predictive analytics; and (iv) lack of clarity about the degree of machine learning-influenced decision-making.^14^ Specific risks for health financing relate to the use of machine learning that facilitates, simplifies or supports actions that are counterproductive to UHC objectives, such as risk selection; cost reductions at the expense of quality of care or financial protection; or excessive monitoring that could create mistrust, for instance by providers towards purchasing agencies. There is a risk that, instead of using machine learning-derived insights to tailor benefits and improve access for underprivileged and vulnerable groups, these insights might be misused to identify patterns in health expenditure and service utilization that exclude certain groups. Using such data could lead to the exclusion of high-cost patients or certain conditions from health coverage, or facilitate raising insurance premiums for high-cost individuals.^1^^,^^18^^,^^19^

For example, a major private health insurance company in South Africa applied machine learning to their customers’ data accrued from supermarket purchases, and activities in fitness firms and health-care use at facilities. This information was used to assess healthy behaviour, with financial rewards, through lower premiums, offered to selected customers.^30^ However, from a health-financing perspective, this approach risks fragmenting the coverage and may discriminate against those most in need of health coverage.

## Guidance and regulations

Whether the effects of machine learning on health financing are positive or negative also strongly depends on how and for which purpose the technology is applied, for instance whether it is used for risk adjustment supporting defragmentation or for risk selection leading to de-solidarization. In addition to general considerations for applying machine learning and artificial intelligence in health care – which include establishing a comprehensive ethical framework, ensuring data governance and data protection, and upholding value-based principles^14^^,^^31^^,^^32^ – it is imperative to implement tailored regulatory measures for health financing. Such regulation is particularly pertinent for voluntary commercial health insurance, considering its potential to fragment health systems and its inherent profit-driven motivations, which could lead to practices aimed at minimizing insurer costs at the expense of broader health system goals.

Regulatory frameworks must mandate clear, accountable procedures that not only secure informed consent from individuals but also clearly disclose the use of health-related data and how they are being used. Equilibrium must be achieved between protecting personal data and fulfilling the legitimate information requirements of purchasing agencies. Furthermore, multisectoral stakeholder consultation processes and governance arrangements are required, in which insurers, regulatory bodies and policy-makers work together for the public interest and system benefits.^18^ Risk mitigation measures could also include algorithm auditing and quality control,^33^ as well as algorithm validation, for example through the analysis of secondary data.

Importantly, algorithms used in health financing need to be presented in such a way that they can be explained and made visible by humans.^29^^,^^34^ Human judgment should be supported and complemented by machine learning, not replaced by it, ensuring that human oversight remains a core standard in the use of artificial intelligence and machine learning.^32^ The need for human oversight is also acknowledged by the European Union’s General Data Protection Regulation, which does not permit insurance claim denials based solely on algorithms.^18^ In a similar vein, meaningful human oversight is a core principle of the Artificial Intelligence and Data Act in Canada; tabled in 2022 and expected to enter into force in 2025 or soon thereafter.^35^

We argue that a more fundamental societal discussion is needed regarding the desirability of artificial intelligence and machine learning in health financing to support the development of regulatory provisions. The recently published WHO guidance note on regulatory requirements related to artificial intelligence in health^36^ will be instrumental in that respect, and can be further operationalized for health financing. 

In Box 2 we propose several key policy and research questions that can be used to inform the development of and discussion about health financing specific guidance, and regulation of ethical and UHC-oriented machine learning use. We assert that multidisciplinary collaboration is required to address these questions by involving health financing policy experts, legal practitioners, artificial intelligence and machine learning specialists, as well as other disciplines. This collaboration should also expand to the design and execution of studies on the effects of artificial intelligence and machine learning as well as the validation of machine learning techniques, since diverse teams will be better positioned to avoid neglecting or underrepresenting the key variables of certain population groups.^29^ To establish and maintain functional collaborative governance platforms, health financing stakeholders must be equipped with the institutional, technical and regulatory capacity, and the adequate human resources to manage and oversee the deployment of artificial intelligence in health financing. There is also a need to create and strengthen algorithmic awareness or algorithmic literacy among health financing system stewards, as well as among citizens, patients, providers and purchasers in relation to health financing.^14^ The list in Box 2 is not exhaustive, and further policy-relevant regulatory and research issues will surely be identified as the field of artificial intelligence and machine learning continues to evolve.

Box 2 **Proposed policy and research questions to guide and regulate the use of machine learning or artificial intelligence in health financing**

***Responsible use of data and big data analytics***

What kind of health financing-related and other governmental or nongovernmental databases should actors be allowed to use and combine? Which data should actors not be allowed to use?

***Managing the capitalization of data***

Which (health financing) actors should have access to health financing-relevant data and under which conditions or requirements? Which health financing stakeholders should be involved in setting the costs to provide access to data? Who should pay for health financing-related data?

***Generating quality data for training and applications of machine learning models***

How to ensure that health financing data is comprehensive and representative to counteract the risk of algorithmic bias and discrimination against individuals or vulnerable population groups (such as low-income groups, women, rural populations) in relation to access to care, quality of care, resource allocation and financial protection?

***Balancing and addressing conflicts of interests in relation to predictive analytics***

What are the legitimate needs and rights of an individual (i.e. the right to not want to know about future health-care needs and costs) weighed against a purchaser’s interests in predictive analytics to support health financing decisions and policy design?

***Exploring limits to the use of machine learning***

Should certain uses of machine learning in health financing and health insurance be restricted, such as for risk selection (e.g. under voluntary health insurance), or for the identification of high-cost patients?

***Determining the desired degree of digitality***

How much artificial intelligence and machine learning-supported decision-making is desirable in the health financing domain, and how much human control should health financing stewards maintain?

***Regulating accountability lines and liability***

Who should be held accountable or liable for errors or harm due to the use of artificial intelligence and machine learning in health financing?

***Setting effective regulatory provisions for the decision-making processes on artificial intelligence and machine learning in health financing***

How should the decision-making, development and revision process of regulatory provisions relating to artificial intelligence and machine learning be organized in view of the speed of technological changes?

***Ensuring the monitoring of machine learning effects***

What kind of information and monitoring systems and processes are needed to ensure that machine learning algorithms have no inequitable or harmful effects in relation to vulnerable population groups (such as low-income groups, women, rural populations)?

The recent emergence of generative artificial intelligence systems based on natural language processing demonstrates this dynamic nature of artificial intelligence and machine learning. In the future, generative artificial intelligence may enter the policy formulation domain and will thus require a fitting government response. Governments will need to regulate the extent to which generative artificial intelligence can be used to support health financing policy development and create machine learning models used for health financing. These regulatory needs constitute non-trivial questions that will have to be answered for all public policy fields including health financing; and more research will be needed.

Whenever the use of machine learning is considered, it is necessary to carefully weigh the benefits and risks to avoid scenarios in which foreseen or unforeseen negative effects outweigh the positive ones. In line with “cautious optimism with safeguards,”^31^ more and rigorous research and systematic evidence should accompany the application of machine learning so as to better understand its effects for health financing oriented towards UHC. We hope that more country-based use cases will be documented and published to inform guidance and policy development. The WHO document *Assessing the effects of digital technologies on health financing and universal health coverage objectives: a guide with key questions* provides guidance in that respect.^17^

In conclusion, only when ethical questions and regulatory issues are properly addressed, will the use of artificial intelligence and machine learning for health financing contribute to the progressive realization of UHC.
